# Characterization of rare spindle and root cell transcriptional profiles in the stria vascularis of the adult mouse cochlea

**DOI:** 10.1038/s41598-020-75238-8

**Published:** 2020-10-22

**Authors:** Shoujun Gu, Rafal Olszewski, Ian Taukulis, Zheng Wei, Daniel Martin, Robert J. Morell, Michael Hoa

**Affiliations:** 1 Auditory Development and Restoration Program, National Institutes on Deafness and Other Communication Disorders, National Institutes of Health, Porter Neuroscience Research Center, 35 Convent Dr., Room 1F-226, Bethesda, MD 20892 USA; 2 Biomedical Research Informatics Office, National Institute of Dental and Craniofacial Research, NIH, Bethesda, MD 20892 USA; 3 Computational Biology and Genomics Core, National Institutes on Deafness and Other Communication Disorders, National Institutes of Health, Bethesda, MD 20892 USA

**Keywords:** Computational biology and bioinformatics, Molecular biology, Auditory system, Computational neuroscience, Ion channels in the nervous system

## Abstract

The stria vascularis (SV) in the cochlea generates and maintains the endocochlear potential, thereby playing a pivotal role in normal hearing. Knowing transcriptional profiles and gene regulatory networks of SV cell types establishes a basis for studying the mechanism underlying SV-related hearing loss. While we have previously characterized the expression profiles of major SV cell types in the adult mouse, transcriptional profiles of rare SV cell types remained elusive due to the limitation of cell capture in single-cell RNA-Seq. The role of these rare cell types in the homeostatic function of the adult SV remain largely undefined. In this study, we performed single-nucleus RNA-Seq on the adult mouse SV in conjunction with sample preservation treatments during the isolation steps. We distinguish rare SV cell types, including spindle cells and root cells, from other cell types, and characterize their transcriptional profiles. Furthermore, we also identify and validate novel specific markers for these rare SV cell types. Finally, we identify homeostatic gene regulatory networks within spindle and root cells, establishing a basis for understanding the functional roles of these cells in hearing. These novel findings will provide new insights for future work in SV-related hearing loss and hearing fluctuation.

## Introduction

**Figure 1:**
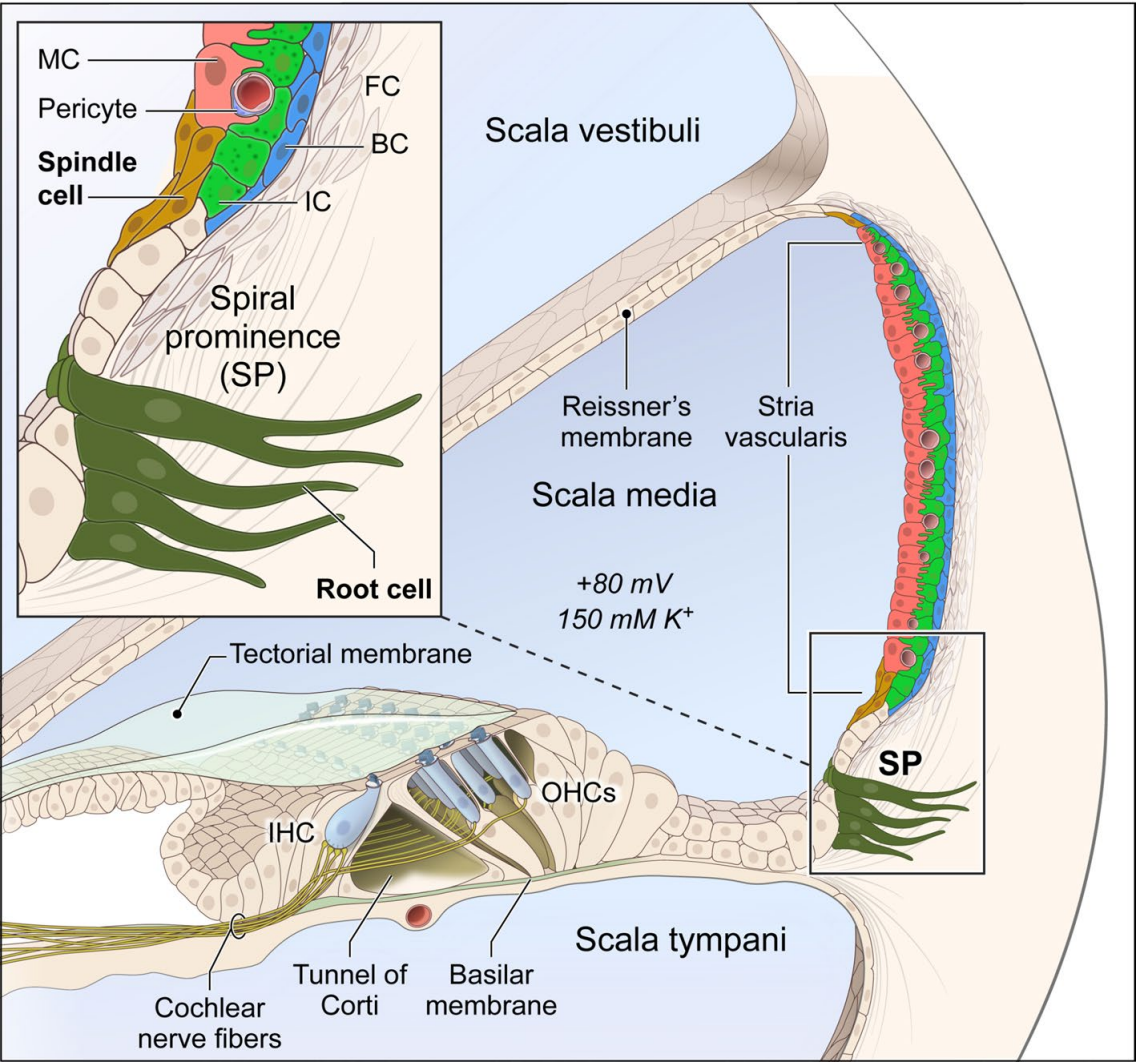
Illustration of adult mouse cochlea. Major stria vascularis cell types (marginal (MC), intermediate (IC) and basal (BC) cells) are colored as red, green and blue, respectively. Rare root cells just outside the stria vascularis and rare spindle cells in the stria vascularis are in magnified view, and colored as dark olive green and dark gold, respectively.

The stria vascularis (SV) is a heterogenous tissue located in the lateral wall of the cochlear duct. It is a stratified epithelium consisting of three major cell types (marginal, intermediate and basal cells), as well as other rare cell types, including spindle cells, macrophages, endothelial cells, and pericytes (Fig. 1). Marginal cells face the endolymph, while the basal cells abut the connective tissue of the spiral ligament. The marginal and basal cell layers sandwich the layer of intermediate cells, which interdigitate with these two cell types^1,2^. These cells work together to produce and maintain the endocochlear potential (EP), which is necessary for normal hearing^3–6^. Mutations in genes expressed in major SV cell types, such as *Kcnq1, Kcne1, Kcnj10* and *Cldn11*, are known to cause deafness and/or SV dysfunction including loss of EP^6–10^. Spindle cells, a rare SV cell type, are found at the superior and inferior borders of the SV and have been implicated in hearing loss and hearing fluctuation^11–13^. Other rare SV cell types including macrophages, pericytes, and endothelial cells^11,14,15^, as well as rare cell types adjacent to the SV including root cells^16,17^, have roles that remain incompletely defined as they relate to EP generation and ion homeostasis in the cochlea. Therefore, understanding the transcriptional profiles and underlying gene regulatory networks in rare SV cell types from the unperturbed adult SV will be critical for mechanistic studies of the role that rare cell types play in EP generation and ion homeostasis in the cochlea.

Our group has previously characterized transcriptional profiles of the major cell types in the adult mouse SV by single-cell RNA-Seq (scRNA-Seq) and single-nucleus RNA-Seq (snRNA-Seq)^2^. However, due to the low number of rare cell types captured, transcriptional profiles for rare SV cell types, specifically spindle and root cells, remained undefined. Previously, we and others have shown that that snRNA-Seq decreases cell size and shape heterogeneity, enabling more comprehensive transcriptional profiling of the tissues with heterogeneity in cell size and shape^2,18–22^. Despite the more comprehensive picture provided by snRNA-Seq, transcriptional profiles of rare SV cell types, specifically spindle and root cells, remained poorly distinguished in our previous study^2^. Recently in other organ systems, sample preservation methods have been utilized to improve capture of rare cell types and we hypothesized that a similar approach might facilitate transcriptional profiling of rare SV cell types^23–25^. In this study, we utilize two sample preservation methods, methanol fixation and RNAlater treatment, in snRNA-Seq to characterize rare cell transcriptional profiles in the adult SV. We define spindle cell transcriptional profiles and distinguish them clearly from those of root cells in the lateral wall of the cochlea. In the process, we compare these methods to the previously published adult SV snRNA-Seq. Finally, we define gene regulatory networks involved in homeostatic functions, referring to functions of the unperturbed adult SV, of spindle and root cells which was not possible with our previously published dataset^2^.

## Results

### Sample preservation process improves snRNA-Seq data quality

**Figure 2:**
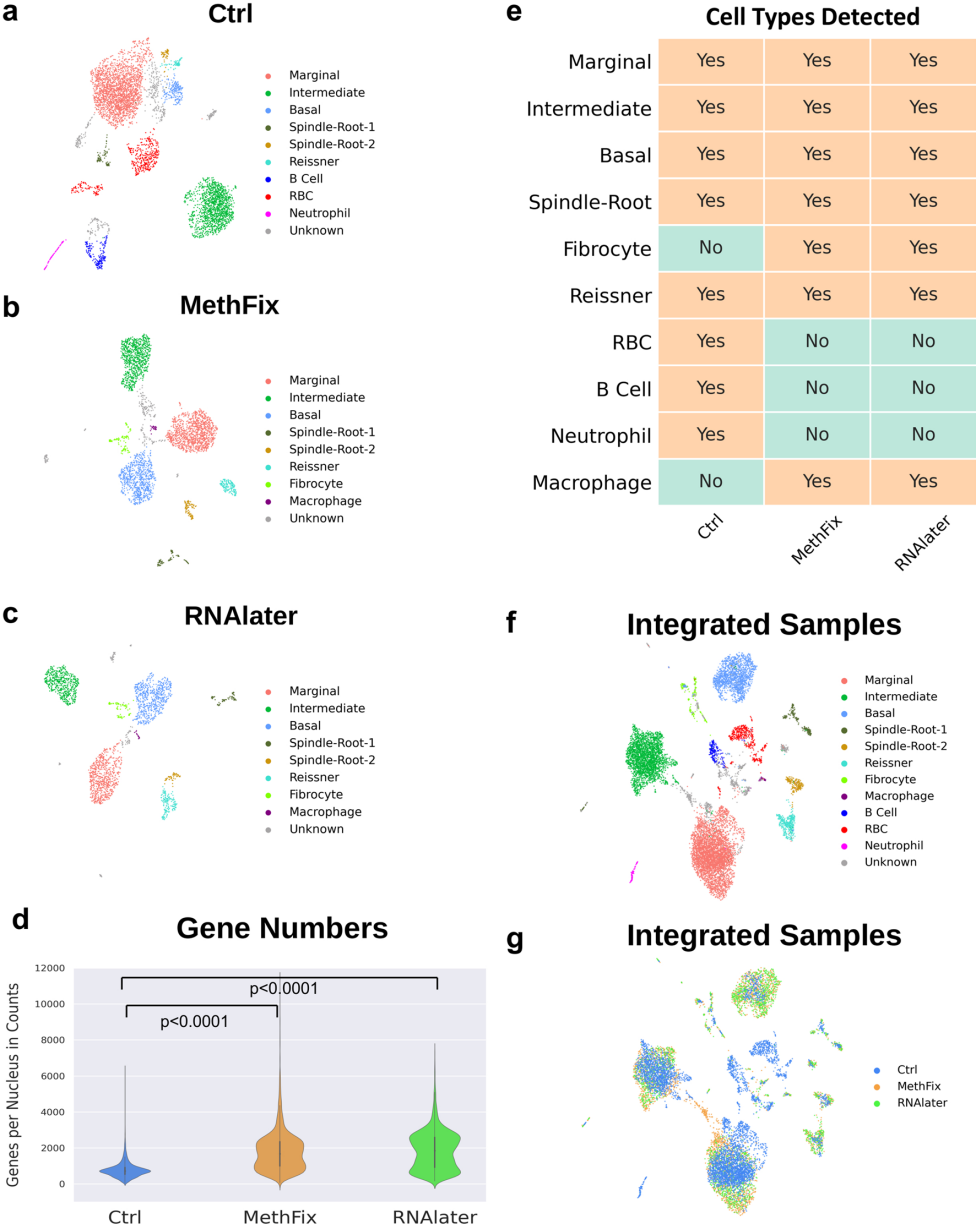
Comparison of Ctrl, MethFix and RNAlater datasets. snRNASeq datasets. (**a**) Ctrl, (**b**) MethFix and (**c**) RNAlater, were clustered by modularity-based clustering method with Leiden optimization algorithm, and visualized by 2D UMAP embedding. (**d**) Increased number of genes detected per nucleus in sample preservation datasets (1682 median genes per nuclei in MethFix, 1857 median genes per nuclei in RNAlater) compared to Ctrl dataset (727 median genes per nuclei) (one-way ANOVA *p* < 0.0001), while no significant difference between MethFix and RNAlater dataset (post hoc t-test, *p* = 0.88). (**e**) Major SV cell types (marginal, intermediate, basal), spindle-root and Reissners’ membrane cells are detected in all three datasets, but other small populations of cells (fibrocyte,macrophage, RBC, B cell and neutrophil ) are detected in either Ctrl or MethFix/RNAlater datasets. Ctrl, MethFix and RNAlater datasets are integrated by Harmony and visualized by 2D UMAP embedding. Integrated cells are colored based on (**f**) cell identities or (**g**) original dataset (Ctrl, MethFix, RNAlater).

As a first step towards analyzing rare cell types in the SV, we utilized two forms of sample preservation, methanol fixation of nuclei (MethFix) and RNAlater treatment (RNAlater) of tissue prior to isolation of nuclei, to generate single nucleus transcriptional profiles from the P30 mouse SV. A total of 5681 nuclei were isolated and analyzed from the P30 mouse SV utilizing these sample preservation methods (3371 nuclei in the MethFix dataset, 2310 nuclei in the RNAlater dataset). We compared our previously published adult SV single-nucleus transcriptional profiles (Ctrl) consisting of 5176 nuclei^2^ to these two datasets (MethFix, RNAlater) (Fig. 2a–c, respectively). The schematic for the different sample preparation steps for all three data sets is shown in Supplementary Figure S1a. Comparison of the three datasets demonstrated an increased number of genes detected per nucleus in the sample preservation datasets with 727, 1682, and 1857 median number of genes per nuclei in the Ctrl, MethFix, and RNAlater datasets (one-way ANOVA *p* < 0.0001). Post hoc t-tests demonstrated a significant difference between Ctrl and either MethFix or RNAlater datasets (both *p* < 0.0001), but not between MethFix and RNAlater datasets (*p* = 0.12) (Fig. 2d). The percentage of nuclei remaining after bioinformatic preprocessing, which included accounting for mitochondrial gene expression and removal of doublet nuclei, was increased in the sample preservation datasets (MethFix, RNAlater) compared to the previously published dataset (Ctrl) (Suppl. Fig. S1b).

### Stria vascularis cell type clusters remain consistent in control and sample preservation snRNA-Seq datasets of the adult SV

Despite these differences, UMAP (Uniform Manifold Approximation and Projection) plots depicting clustering for the previously published adult SV dataset (Fig. 2a)^2^ and the two datasets where sample preservation methods was employed (MethFix, RNAlater) (Fig. 2b,c, respectively) demonstrated similar clusters of SV cell types. SV cell type clusters including marginal, intermediate, basal, spindle/root cells, as well as, cells of Reissner’s membrane, were identified in all 3 datasets (Ctrl, MethFix, RNAlater). Furthermore, other small populations of cells including fibrocytes and macrophages were identified as unique clusters in both sample preservation datasets (MethFix, RNAlater) (Fig. 2b,c). In contrast, red blood cells (RBCs), B cells, and neutrophils were only detected in the Ctrl dataset (Fig. 2a). A summary of cell types identified in the three datasets is shown (Fig. 2e).

To understand how the proportions of cell types detected in each of the datasets (Ctrl, MethFix, RNAlater) reflected in vivo proportions, we compared the relative percentages of major SV cell types (marginal, intermediate, basal, and spindle/root cells) in all three datasets to in vivo percentages calculated from cell counts from mid-modiolar cochlear cross-sections (Suppl. Fig. S1c-f). Relative in vivo percentages of SV marginal, intermediate, basal and spindle/root cells (N = 6 adult mice) are shown with a representative mid-modiolar cochlear cross-section of the SV (Suppl. Fig. S1c and S1d, respectively). By comparison to the Ctrl dataset (Suppl. Fig. S1e), sample preservation datasets (Suppl. Fig. S1f. and S1g) demonstrated percentages of major SV cell types that were more similar to in vivo major SV cell type percentages (Suppl. Fig. S1c). Furthermore, the percentage of rare spindle/root cells in the MethFix and RNAlater datasets were 6.2% and 6.3%, respectively, nearly doubling the 3.4% of these cells in the control dataset.

In order to visualize SV cells from all three datasets, the datasets were integrated with batch correction utilizing Harmony as previously described^26,27^. All SV cell types remained clustered together with cell identities based on clustering of individual datasets (Fig. 2f). The distribution of the three datasets (Ctrl, MethFix, RNAlater) within each of the clusters is shown in Fig. 2g. Major SV cell types remained in cell type-specific clusters after dataset integration. The expression pattern of representative marker genes used for cluster labeling are shown in Supplementary Figures S2-1 and S2-2. While, fibrocyte gene (*Coch*) expression was detected in the Ctrl dataset (Suppl. Fig. S2-2), these cells partially overlapped with other SV cell type clusters and as a result were not annotated as a fibrocyte cluster in the Ctrl dataset. Interestingly, in all three P30 SV data sets, two sub-clusters of spindle-root cells are revealed, and they are labeled as Spindle-Root-1 and Spindle-Root-2, respectively.

### Differential expression (DE) analysis reveals transcriptional differences between rare populations of spindle and root cells

**Figure 3:**
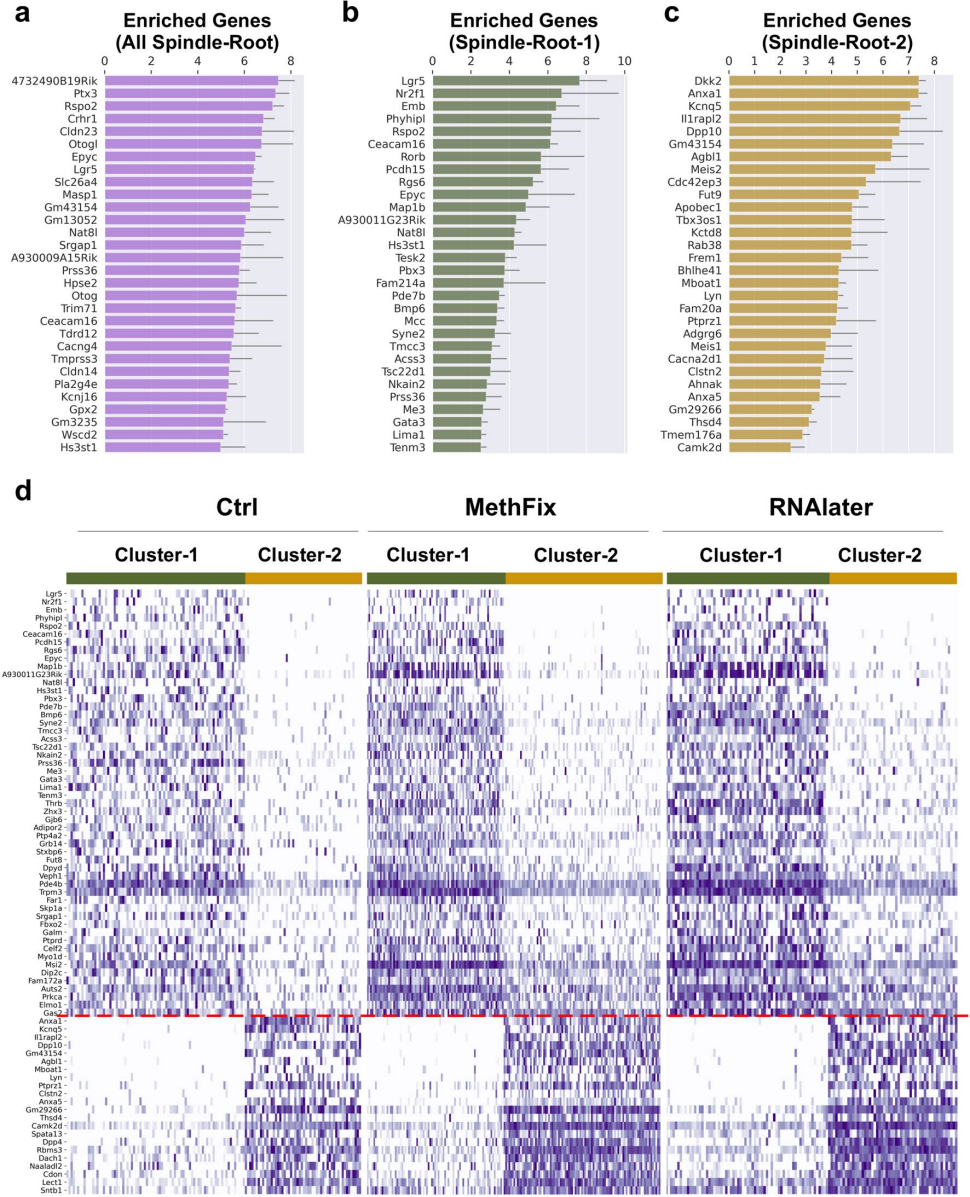
Differential expression analysis on Ctrl, MethFix and RNAlater datasets. Top 30 enriched genes in (**a**) spindle-root cells compared to other cell types, (**b**) Spindle-Root-1 compared to Spindle-Root-2 and (**c**) Spindle-Root-2 compared to Spindle-Root-1 are shown in barplot. The logarithm (base 2) of the fold-change (logFC) are displayed along the horizontal axis, and gene names are displayed along the vertical axis. Standard deviation of logFC among three datasets is shown with the error bar. (**d**) Normalized counts of unique enriched genes in Spindle-Root-1 (above red dashed line) and Spindle-Root-2 (below red dashed line) are shown as heatmaps for each dataset (Ctrl, MethFix, RNAlater). Nuclei are displayed along the horizontal axis and genes are displayed along the vertical axis.

Cell type-specific gene expression across all three snRNA-Seq datasets for SV cell types (Ctrl, MethFix, RNAlater) is shown in Fig. 3. The top 30 up-regulated genes (*p* value < 0.05, and sorted by fold change) in Spindle-Root cells obtained by comparing to other cell types are listed in Fig. 3a. Mean fold-change values were calculated based on DE analysis determined within each dataset (Ctrl, MethFix, RNAlater). Among the top 30 up-regulated genes in Spindle-Root cells were *Slc26a4* and *Kcnj16*, which are expressed in both sub-clusters of Spindle-Root cells and is consistent with our previously published spindle-root cell clustering^2^. Based on the observation of two sub-clusters of Spindle-Root cells, we took advantage of this opportunity to perform a more detailed analysis of the transcriptional differences between the Spindle-Root sub-clusters. The top 30 significant (*p* < 0.05) up-regulated genes in each Spindle-Root sub-cluster are shown in Fig. 3b,c, respectively.

To identify uniquely expressed genes in the each of the Spindle-Root sub-clusters, the DE gene lists for Spindle-Root-1 (Fig. 3b) and Spindle-Root-2 (Fig. 3c) were cross-referenced against genes expressed by other SV cell types to identify differentially expressed genes expressed by Spindle-Root-1 and Spindle-Root-2 (Fig. 3d). The resulting list of DE genes were compared across all 3 datasets and the list of commonly expressed genes between the 3 datasets were displayed in heatmaps for the Ctrl (Fig. 3d, left), MethFix (Fig. 3d, middle), and RNAlater (Fig. 3d, right) datasets. The resulting lists of differentially expressed genes demonstrate that the Spindle-Root-1 and 2 exhibit distinct gene expression patterns.

**Figure 4:**
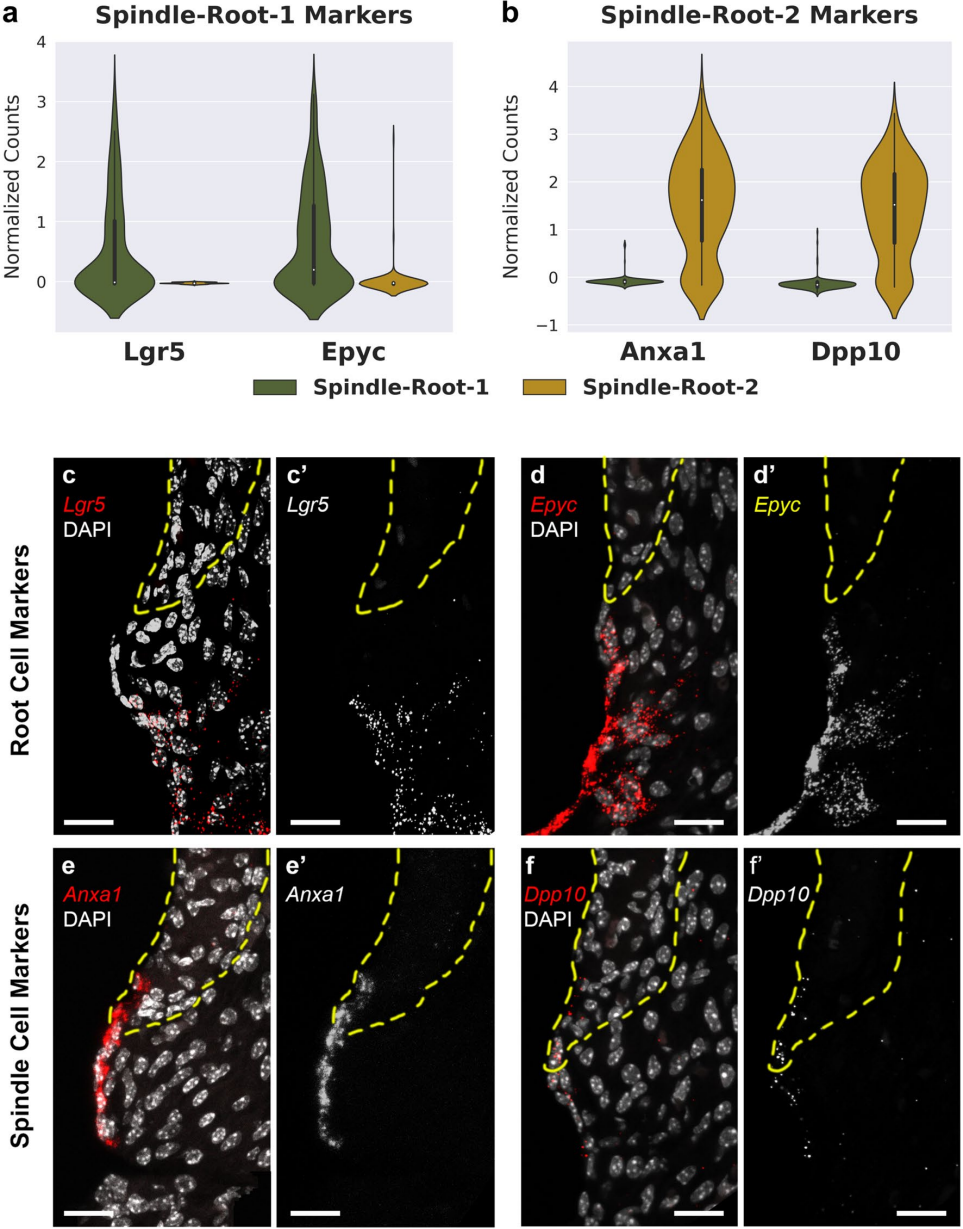
Validation of identified markers in Spindle-Root sub-clusters by single-molecule fluorescent in situ hybridization. Expression level (normalized counts) of (**a**) Spindle-Root-1 candidate markers (*Lgr5* and *Epyc*) and (**b**) Spindle-Root-2 candidate markers (*Anxa1* and *Dpp10*) are shown in violin plots. (**c**–**d’**) smFISH demonstrates expression of *Lgr5* (**c**, **c’**) and *Epyc* (**d**, **d’**) in root cells, while expression of *Anxa1* (**e**, **e’**) and *Dpp10* (**f**, **f’**) are detected in spindle cells. Grayscale images of smFISH probe are shown in single channel images (**c’**–**f’**). Scalebars are all 20 μm. Yellow dotted lines indicate location of stria vascularis. DAPI labels cell nuclei.

To investigate the expression pattern of identified Spindle-Root sub-cluster markers in Fig. 3, select candidate genes were visualized on violin plots and single-molecule fluorescent in situ hybridization (smFISH) was performed for selected marker genes (Fig. 4). Violin plots, demonstrating expression level in normalized counts, for Spindle-Root-1 candidate genes, Leucine Rich Repeat Containing G Protein-Coupled Receptor 5 (*Lgr5*), and Epiphycan (*Epyc*) are shown in Fig. 4a. Violin plots for Spingle-Root-2 candidate genes, Annexin A1 (*Anxa1*) and Dipeptidyl Peptidase Like 10 (*Dpp10*), are shown in Fig. 4b. Interestingly, we found that Spindle-Root-1 enriched genes *Lgr5* (Fig. 4c, c’) and *Epyc* (Fig. 4d, d’) are expressed in root cells, while Spindle-Root-2 enriched genes *Anxa1* (Fig. 4e, e’) and *Dpp10* (Fig. 4f, f’) are expressed in SV spindle cells. Since KCNJ10 protein has been previously shown to be expressed in root cells^16,17^ in addition to SV intermediate cells^2,9,28^, we co-localized *Lgr5* RNA with *Kcnj10* RNA in root cells (Suppl. Fig. S3a, a’). While *Anxa1* was expressed by the spiral prominence surface epithelial cells in continuity with the spindle cells, we did not identify a distinct transcriptional cluster of these surface epithelial cells in the spiral prominence. Neither *Anxa1* nor *Dpp10* RNA was expressed in other regions of the cochlea (data not shown). *Lgr5* RNA expression was also observed in Deiters cells and cells of the outer sulcus (Suppl. Fig. S3b). In addition to root cells, *Epyc* RNA expression was localized to the outer sulcus and the greater epithelial ridge (Suppl. Fig. S3c). smFISH validation of *Lgr5* and *Epyc* expression (Fig. 4c,d, respectively) appears to be consistent with previous publications that demonstrated expression of these genes in the region of the outer sulcus or spiral prominence^29,30^. Therefore, our data demonstrate that Spindle-Root cells can be further distinguished into spindle and root cell clusters by previously uncharacterized marker gene expression. Based on marker gene expression confirmed by smFISH, Spindle-Root-1 cells and Spindle-Root-2 cells will be referred to as root and spindle cells, respectively.

To determine the difference between the three datasets, we combined the datasets on their mutual genes without applying any data merging algorithm. A detectable batch effect, as demonstrated by the cells from each dataset clustering into distinct clusters, was observed between the Ctrl data set and the MethFixed (Suppl. Fig. S4a) and RNAlater (Suppl. Fig. S4b) data sets, respectively. However, a batch effect was not detected between MethFix and RNAlater datasets as suggested by the overlapping distributions amongst cell types between the two sample preservation datasets (Suppl. Fig. S4c) as well as when all three datasets are combined together (Suppl. Fig. S4d). Comparison of the combined MethFix and RNAlater datasets without batch correction (Suppl. Fig. S4e) to the combined MethFix and RNAlater datasets integrated with batch correction using Harmony as previously described^26,27^ demonstrated minimal impact on the clustering of SV cell types (Suppl. Fig. S4f.). Therefore, MethFix and RNAlater datasets were combined (MethFix-RNAlater) (Suppl. Fig. S4e) for downstream gene regulatory network analysis without using additional data merging algorithms.

### Gene regulatory network landscape in SV root and spindle cells

**Figure 5:**
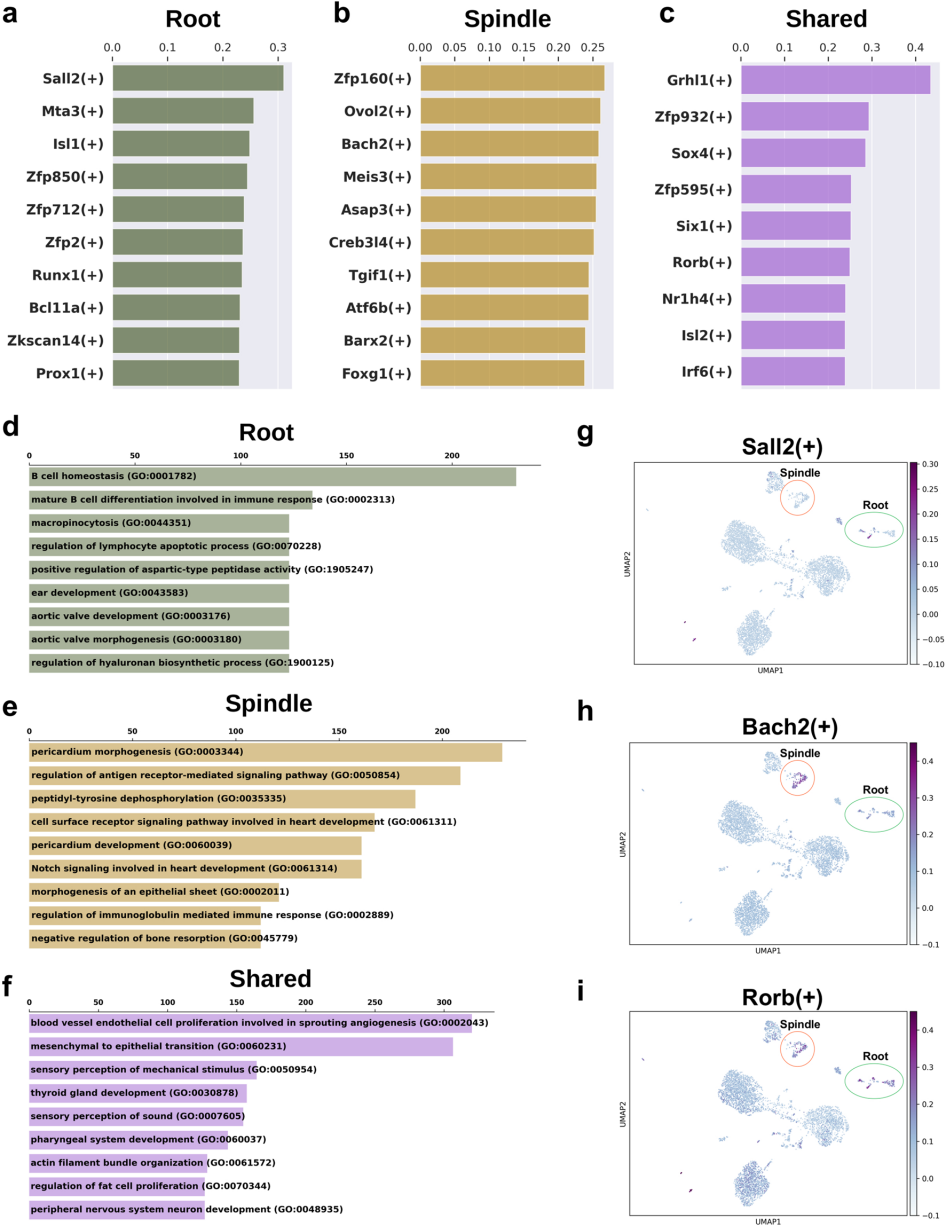
Regulon analysis by pyScenic on combined MethFix-RNAlater dataset. Top non-shared regulons by regulon specificity score (RSS) for (**a**) root cells and (**b**) spindle cells and (**c**) Top shared regulons are shown in bar plots. X-axis is the RSS. The top enriched GO biological process terms for genes in (**d**) the top 10 non-shared root regulons, (**e**) the top 10 non-shared spindle regulons and (**f**) the top shared regulons are ranked by combined score from Enrichr. Regulon activity (AUC score) of (**g**) Sall2, (**h**) Bach2 and (**i**) Rorb are shown in the 2D UMAP of MethFix-RNAlater combined dataset. Root and spindle clusters are highlighted by green and red circle, respectively.

To further explore potential homeostatic functional differences and similarities between spindle and root cells, we applied SCENIC as previously described by our labs and others^2,31^ to the combined sample preservation (MethFix-RNAlater) P30 mouse SV datasets (Suppl. Fig. S4c), which demonstrated overlapping distributions amongst cell types clusters without batch correction (Suppl. Fig. S4e). The top 10 regulons for root and spindle cells as determined by the regulon specificity score (RSS) along the horizontal axis are shown in Fig. 5a,b, respectively. The top shared regulons between root and spindle cells are shown in Fig. 5c. The higher the RSS, the more specific a given regulon is to the spindle and root cells, respectively^32^. The top enriched GO biological process terms for root, spindle, and shared root and spindle regulons were B cell homeostasis (GO:0001782), pericardium morphogenesis (GO:0003344), and blood vessel endothelial cell proliferation involved in sprouting angiogenesis (GO:0002043), respectively (Fig. 5d–f). *Sall2* and *Bach2* regulon activity are shown for root and spindle cells, respectively (Fig. 5g,h, respectively). Both regulons represent novel gene regulatory networks that have not been related previously to homeostatic function in the inner ear. Putative *Sall2* target genes include *Sall2* itself and *Nr2f1* with UMAP plots in Supplementary Figure S5 (Suppl. Fig. S5a and S5b, respectively) demonstrating expression in root cell predominantly. *Nr2f1* also expressed by cells of Reissner’s membrane and an unknown population of cells in close proximity to root cells on the UMAP plot. While *Bach2* has been previously shown to be expressed in chick otic epithelium^33^ and is known for its role in the *Bcl6-Bcl2-p53* axis which controls hair cell apoptosis (reviewed by Morill and colleagues^34^), its role in the inner ear, either in inner ear development or in hearing remain incompletely characterized. Expression of putative *Bach2* target genes including *Anxa1* and *Dpp10* are shown with UMAP plots (Suppl. Fig. S5c and S5d, respectively) and are validated by smFISH (Fig. 4e–e’ and f–f’) and colocalized with *Bach2* (Suppl. Figure 6a–a’).

The *Rorb* regulon, which is shared between root and spindle cells, and its regulon activity plot are shown in Fig. 5i. Putative *Rorb* target genes include *Otog*, *Cldn14*, and *Pde4b*. While mutations in *Otog*^35,36^ and *Cldn14*^37–39^ have been linked to hearing loss, mutations in *Pde4b* has not been previously linked with hearing loss. UMAP plots of gene expression for *Rorb*, *Otog*, *Cldn14*, and *Pde4b* are provided in the supplement (Suppl. Fig. S5e-h, respectively). While *Rorb* is expressed in the region of the future root cells as well as the organ of Corti in the apical cochlea at E15.5 with expression decreasing towards the basal turn of the cochlea, its expression is noted to be more widespread in the adult mouse cochlea^40^. Our data identifies *Rorb* expression in both root and intermediate cells of the adult SV (Suppl. Fig. S5e). *Otog* expression (Suppl. Fig. S5f) is consistent with previously published expression in root cells in the perinatal mouse cochlea^35^. *Cldn14* expression (Suppl. Fig. S5g) is seen in both root and spindle cells of the adult SV and is consistent with previously reported expression by Ben-Yosef and colleagues^37^. While not previously described in the inner ear, *Pde4b* is expressed by both root and spindle cells as well as intermediate cells of the adult SV (Suppl. Fig. S5h).

## Discussion

In this study, we utilize two sample preservation methods with snRNA-Seq to characterize rare cell transcriptional profiles in the adult SV. We compare these transcriptional profiles to previously published adult snRNA-Seq, demonstrating not only comparability, but also demonstrate the advantages of sample preservation in increasing the yield of rare cell types. Compared to our previous study which focused on transcriptional profiles of major SV cell types including marginal, intermediate and basal cells^2^, this current study utilizes novel methodologies to identify transcriptional profiles of rare SV cell types, notably spindle and root cells. While the small numbers of these rare cell types in our previously published control single nucleus dataset^2^ limited resolution of their transcriptional identities, we hypothesized that sample preservation methods might enable isolation of greater numbers of these particular rare cell types to resolve their transcriptional profiles. One caveat is that different sample isolation methods may result in preferential capture of certain cell types, necessitating that methods be tailored to the objectives of a given study^24^. Notably, Denisenko and colleagues noted that immune cells were detected at lower rates across all single nucleus RNA-sequencing experiments^24^. While the capture of circulating immune cell types including B cells and neutrophils was less robust with these sample preservation methods, our goal of isolating rare intrinsic cell types of the SV and adjacent tissues including spindle and root cells was achieved. In addition, sample preservation methods also allowed the capture of other rare cell types including a larger number of fibrocytes and macrophages, which were not detected in the control snRNA-Seq dataset.

We demonstrate that use of RNAlater as a sample preservation method for snRNA-Seq is viable and comparable to methanol fixation. Sample preservation techniques led to a higher percentage of rare cell types by the near doubling of root and spindle cells captured while the median genes per nuclei was increased in the sample preservation datasets compared to control dataset. Alterations in our bioinformatic processing pipeline (see Supplementary Note) from our published analysis^2^ may have contributed to the ability to resolve rare cell populations. While use of methanol fixation is employed after nuclei isolation and requires fairly rapid isolation of nuclei from tissue, the use of RNAlater allows for the placement of tissue at room temperature allowing for some flexibility in tissue and nuclei isolation. These results suggest that this method might be utilized with precious difficult-to-obtain tissue (i.e. human pathological tissue) for the purposes of snRNA-Seq, potentially facilitating collaborations across institutions, leading to further application of these technologies to human disease. Furthermore, regulatory network identification for spindle and root cells was not possible with the control dataset and was entirely based on data derived from the sample preservation datasets possibly due to the higher percentage of rare cell types captured. While these newly identified potential regulatory networks will need to be validated, examined and perturbed in future studies, our study establishes a basis for perturbing these regulatory networks.

Despite work elucidating the cyto-architecture and structural components involved in membrane physiology of root cells reviewed by Jagger and Forge^17^, the functional roles of root cells remain largely undefined. Proposed functions for root cells include cation absorption from the endolymph, potassium transport between junctional compartments, and involvement in the inflammatory response of the cochlea to pathological stress^16,17^. The involvement of root cells in cochlear ionic homeostasis is supported by previously published root cell expression of the α1 and β1 subunits of the Na, K-ATPase antiporter in the rat^41^, expression of the AE2 Cl^−^/HCO_3_^−^ exchanger in root cell processes invading the spiral ligament in the guinea pig^42^, expression of SLC26A4, an anion exchanger for chloride and bicarbonate, in the root cell processes in the adult mouse^43^ in addition to its expression in spindle cells^12^, and the expression of Kir4.1, encoded by *Kcnj10*, in root cells^16^ in addition to its expression in intermediate cells in the SV^2,3,44^.

Neonatal and adult *Epyc* knockout mice, while possessing normal-appearing cochleae including hair cells and supporting cells, have elevated hearing thresholds above 16 kHz on ABR, suggesting a role for *Epyc* in hearing^30^. Despite work that has established *Lgr5*, a cell membrane receptor of the Wnt signaling pathway, as a marker of potential inner ear stem cells^45–49^, its role in hearing is uncertain. Both *Lgr5* and its ligand, R-Spondin 2 (*Rspo2*), are differentially expressed by root cells when compared to spindle cells (Fig. 3). *Rspo2* has been shown to promote clustering of acetylcholine receptors through its interaction with *Lgr5* at the neuromuscular junction^50^ and the expression of acetylcholine receptors has been previously demonstrated in the region of the root cells^51^. While the role of these acetylcholine receptors is generally thought to be inhibitory^51^, their role is largely undefined. Wangemann and colleagues have previously shown that potassium secretion in SV marginal cells is negatively regulated by stimulation of muscarinic acetylcholine receptors^52^, suggesting the possibility that *Lgr5* and its ligand, *Rspo2*, may play some role in regulating the undefined function of these receptors in root cells. Jagger and Forge have suggested that root cells “act as a continuous K^+^ sink” likely from the endolymph and we suggest that the ability to inhibit potassium entry may be protective by possibly preventing potassium loss from the endolymph in situations where potassium homeostasis is disrupted in the endolymph^17^. However, this is highly speculative with the functional roles in root cells of *Lgr5* and *Rspo2* remaining largely undefined. Our study establishes a possible rationale for future experiments which will define the functional roles of these genes in root cells.

Similar to root cells, the role of spindle cells in hearing have remained poorly defined. In this study, DE analysis identifies spindle cell-specific transcriptional profiles for the first time and validation of two candidate genes, *Anxa1* and *Dpp10*, consistently distinguishes these cells from adjacent root cells (Fig. 4e,f). *Dpp10* is a previously uncharacterized transmembrane channel protein in the inner ear, with an undefined role in SV function and hearing, that may play a role in modulating the activity of voltage-gated potassium channels^53^. While *Anxa1* RNA expression in other regions of the organ of Corti notably Hensen cells (data not shown) as described by Kalinec and colleagues^54^ was not seen, the existence of secreted forms and observations of ANXA1 in punctate form around lipid droplets leave open the possibility that other cochlear cell types may store ANXA1 after production. Alternatively, glucocorticoid stimulation could result in production of *Anxa1* in other cochlear cell types not included in our unstimulated snRNA-Seq datasets. *Anxa1* expression by smFISH (Fig. 4e,e’) in spindle cells and the surface epithelial cells of the spiral prominence suggests that these cell types, if distinct, may share transcriptional similarities. Alternatively, it is possible that a limited collection of these spiral prominence surface epithelial cells in our dataset limits the ability to transcriptionally distinguish them. Nonetheless, *Anxa1* expression distinguishes spindle cells from root cells.

Closer examination of spindle cell-specific gene regulatory networks supports the potential role of spindle cells in responses to inflammation. Specifically, the *Bach2* regulon (Fig. 5h), a spindle cell-specific regulon, includes *Anxa1* and *Dpp10*. *Bach2* is a transcription factor belonging to the BTB and Cap’n’collar (CNC) gene family that functions within multiple innate and adaptive lineages to control immune response. *Bach2* functions as a transcriptional repressor and is a known susceptibility gene for a number of autoimmune diseases^55–57^. *Bach2* appears to promote a shift from myeloid to lymphoid programs by suppressing myeloid genes in B cells^58^ as well as directing T helper (Th) cell differentiation, homeostasis, and effector functions while preventing full effector differentiation within Th cells in vitro^57^. Aberrant expansion of T follicular helper (Tfh) cells results in pathogenic autoantibodies and is frequently associated with autoimmune diseases^57,59,60^. Recently, Zhang and colleagues demonstrated that the loss of *Bach2* expression led to increased Tfh cell accumulation with a shift towards an IL-4-producing subset^57^. These data suggest that *Bach2* prevents humoral autoimmunity, at least in part by inhibiting the generation of pathogenic Tfh cells. *Bach2* also appears to regulate differentiation and effector functions of other T cell subsets including Treg and Th17 cells, which play prominent roles in autoimmunity when deregulated^61,62^. Finally, *Bach2* appears to be necessary for an appropriate macrophage responses to T cell-induced inflammation^63^. Thus, *Bach2* regulon activity in spindle cells suggests that these cells may play a role in regulating autoimmune responses in the inner ear. Furthermore, gene regulatory networks in root cells corroborate the suggested role of root cells in endolymph ion homeostasis as reviewed by Jagger and Forge^16,17^. The *Sall2* regulon (Fig. 5g), a root cell-specific regulon, includes target genes *Nr2f1* and *Sall2*. While *Sall2* has not previously been characterized in the inner ear, mutations in *Nr2f1* result in hearing loss^64–66^. *Nr2f1* expression is present in the cells which are fated to become the root cells in the cochlea^66,67^. Tarchini and colleagues demonstrate that long-range downregulation of *Nr2f1* through a mutation in *Mctp1* in the *deaf wanderer* (*Mctp1*^*dwnd*^) mice results in hearing loss with minimal alterations to cochlear structure^67^. Intriguingly, Bergeron and colleagues demonstrate that overexpression of *Nr2f1* in the Spot^Tg/Tg^ mutant mouse results in expansion of the endolymph-containing scala media with melanocytes failing to migrate to their proper locations in the vestibule but not the cochlea^68^. Despite, the apparent absence of hair cell degeneration in the face of *Nr2f1* downregulation, hearing loss associated with changes in the endolymph ion homeostasis may occur in a delayed fashion^69–71^. The difficulty with Spot^Tg/Tg^ mutant mouse model is that the vast majority of offspring die at birth with few that survive to young adulthood, thus making auditory testing inconclusive. Nonetheless, in combination with previously published expression of ion channel proteins expressed by root cells, including Na, K-ATPase antiporter, AE2 Cl^−^/HCO3^−^ exchanger, SLC26A4, and Kir4.1^17,41–43^, our observations of root cell regulons corroborate the idea that root cells may be involved in regulation of endolymph ion homeostasis.

Finally, shared root and spindle cell regulons implicate these cells in Meniere’s disease, an inner ear disease with a poorly understood pathophysiology and with no implicated cell types^72–74^. The retinoid-related orphan receptor β (*Rorb*) regulon is a shared between root and spindle cells. *Rorb* expression has been previously demonstrated to be involved in the differentiation of neuronal cell types and in regulating circadian activity^75–77^. In addition to previously validated expression of *Slc26a4*^2^, spindle and root cells express potential *Rorb* target genes, *Otog* and *Cldn14* (Suppl. Figure 5f and g, respectively). Recently, missense mutations in *Slc26a4*, *Otog*, and *Cldn14* have been characterized in patients with Meniere’s disease^38,78,79^. These authors suggest that acquisition of additional mutations over time in the form of missense mutations may underlie the onset of Meniere’s disease. Single cell transcriptional data provides an opportunity to identify potentially involved cell types in human disease^80^. This is particularly relevant to diseases where the organ of interest is difficult to access and has rare opportunities for tissue sampling in humans, as is the case for the inner ear. Thus, these data suggest that root and spindle cells may be involved in the pathophysiologic mechanisms underlying Meniere’s disease.

In conclusion, we define distinct transcriptional profiles for rare SV spindle cells and root cells in the spiral prominence. We characterize putative gene regulatory networks for these rare cell types and in doing so, identify potential roles that these cell types may play in the cochlea, including ion homeostasis and regulation of immune responses in the cochlea. Furthermore, we implicate these rare cell types in both genetic and acquired hearing loss. Finally, we provide some initial evidence that dysfunction in spindle and root cells may be related to Meniere’s disease.

## Methods

### Animal model and experimental design

CBA/J mice were purchased from JAX (Stock No. 000656). Postnatal day 30 (P30) mice were used for snRNA-Seq experiments and single molecule RNA fluorescent in situ hybridization (smFISH).

### Adult mouse stria vascularis preparation

The method of adult mouse SV preparation has been previously described^2^. Briefly, the lateral wall of the cochlea was microdissected from the bony wall of adult mouse cochlea and the pigmented strip in the cochlea lateral wall denoting the SV was microdissected from the spiral ligament using fine forceps. SV from all turns of the cochlea were collected. Samples were collected at the same time of day across individual mice and batches. For each collection, less than 1 h was spent prior to single nucleus capture on the 10 × Genomics Chromium platform. Sexes of mice were generally mixed for each experiment. 5 mice (2 female, 3 male P30 mice) were used for the methanol-fixed single nucleus capture and 6 mice (3 female, 3 male P30 mice) were used for the RNAlater-treated single nucleus capture. For the methanol-fixed sample, isolated cell nuclei obtained as previously described^2,23,81^. Briefly, nuclei were suspended in 200 μL Dulbecco’s phosphate buffered saline (DPBS), then 800 μL of ice-cold methanol was slowly added drop-by-drop to the single nuclei suspension while gently stirring the nuclei suspension. Nuclei were moved to the freezer and incubated 30 min at − 20 °C. Subsequently, cells were rehydrated in wash and resuspension buffer (1 × PBS with 1% BSA and 0.2 U/ul RNase Inhibitor). Nuclei suspension underwent centrifugation (100 rcf, 5 min, 4 °C) and supernatant was removed and cells were resuspended in 50 μL of wash and resuspension buffer to obtain 700–1200 cells/μL prior to nuclei isolation and sequencing. For the RNAlater-treated sample, freshly dissected adult SV tissues were submerged in and stored in 0.7 mL of RNAlater solution (Catalog No. AM7020, ThermoFisher, Waltham, MA) at room temperature in a 1.5 mL Eppendorf tube and then stored at 4 °C overnight. After incubation, DPBS was added in equal volumes (0.7 mL) to the tube and gently mixed, then centrifuged at 500 g for 5 min at room temperature. Supernatant was removed and replaced with lysis buffer before previously described nuclei isolation and sequencing.

### Single nucleus suspension

Isolation of nuclei from the adult mouse SV has been previously described^2^. Briefly, SV from ~ 5 to 6 P30 animals were isolated and collected in 3 ml DMEM F-12 media. Following collection, the media was replaced with 3 ml chilled lysis buffer (10 mM Tris-HCl, 10 mM NaCl, 3 mM MgCl_2_, 0.005% Nonidet P40 in Nuclease free water) and the tissue were lysed at 4 °C for 25 min. The lysis buffer was then replaced with 1.5 ml DMEM F-12 media. The tissues were triturated and filtered through a 20um filter (pluriSelect Life Science, El Cajon, CA). The filtrate was centrifuged at 500rcf for 5 min at 4 °C. The supernatant was removed, and the cell pellet was resuspended in 1 ml nuclei wash and resuspension buffer (1 × PBS with 1% BSA and 0.2 U/μl RNase Inhibitor). The cells were filtered through a 10um filter (pluriSelect Life Science, El Cajon, CA) and centrifuged at 500rcf for 5 min at 4 °C. The supernatant was removed, and pellet resuspended in 50 μl of nuclei wash and resuspension buffer. Nuclei were counted in a Luna cell counter (Logos Biosystems, Annandale, VA) and a nuclear density of 1 × 10^6 ^cells/ml was used to load onto the 10× genomics chip.

### 10× Chromium genomics platform

Single nuclei captures were performed following manufacturer's recommendations on a 10× Genomics Controller device (Pleasanton, CA). The targeted number of captured nuclei ranged from 6000 to 7000 per run. Library preparation was performed according the instructions in the 10× Genomics Chromium Single Cell 3′ Chip Kit V2. Libraries were sequenced on a Nextseq 500 instrument (Illumina, San Diego, CA) and reads were subsequently processed using 10× Genomics CellRanger analytical pipeline using default settings and 10× Genomics downloadable mm10 genome as previously described^2^.

### Single-nucleus RNA-seq data preprocessing

*Quality Control*—snRNA-Seq data preprocessing was conducted by Scanpy (v1.4.5)^82^. Genes were filtered based on number of cells. Only genes detected in at least 3 cells are kept. Low-quality cells were filtered out when: (1) less than 200 genes were detected; (2) more than 8000 counts in total; and (3) more than 10% of mitochondria genes were detected.

*Doublet detection*—Transcriptional profiles of doublet nuclei in the snRNA-Seq dataset were computationally predicted by Scrublet (v0.2.1)^83^, and excluded from downstream analysis. Default parameters were utilized including an expected doublet rate of 0.1, number of principle components set to 30, and minimal gene variability set to 85.

### Clustering and data visualization

To cluster the cells by their expression, we used modularity-based clustering with Leiden algorithm implemented in Scanpy (v1.4.5) for each dataset separately. In brief: (1) raw counts were normalized by total with parameter *exclude_highly_expressed* set as True, and scaled by the function *pp.log1p*; (2) principal component analysis (PCA) was performed on top 4000 high variable genes, which were generated with default threshold of the mean expression and dispersion by the function *pp.highly_variable_genes*; (3) KNN graph was constructed based on the euclidean distance in top 30 PCA dimensions by the function *pp.neighbors* with parameter *n_neighbors* set as 10; (4) Cells were clustered by the function *tl.leiden* with the resolution Ctrl = 1.0, MethFix = 1.0 and RNAlater = 1.5. Clustered cells were visualized by Uniform Manifold Approximation and Projection (UMAP) embedding with 2 components. Heatmaps or violin plots were constructed as previously described^2^.

### Dataset integration

The integration of all three datasets (Ctrl, MethFix, RNAlater) and the sample preservation datasets (MethFix, RNAlater) was performed by utilizing the Harmony algorithm, which projects cells into a shared embedding^26^. The package used for data integration was harmony-pytorch (v0.1.3). Default parameters were utilized.

### Cell cycle and dissociation effect calibration

Biological effect calibration is conducted by Scanpy (v1.4.5).

*Cell cycle effect calibration*—Cell cycle heterogeneity in snRNA-Seq data sets was calibrated by calculating cell cycle phase scores based on identified cell cycle markers as previously described^84^. Briefly, cell cycle phase score is calculated by the difference of mean expression of the list of cell cycle genes^85^ and the mean expression of reference genes. The reference genes are randomly selected, which match the distribution of the expression of the given list. Detailed steps can be found in Scanpy tutorial documents (https://nbviewer.jupyter.org/github/theislab/scanpy_usage/blob/master/180209_cell_cycle/cell_cycle.ipynb).

*Dissociation effect calibration*—Dissociation effect calibration in snRNA-Seq data sets was performed similarly to cell cycle effect calibration based on identified dissociation related genes^86^. We have previously detailed the application of this technique to the single nucleus RNA-Seq data from the adult SV^2^. Cell cycle and sample dissociation effects minimally impact cluster composition in major SV cell types (Supplementary Note, Suppl. Fig. S7).

### Differential expression (DE) analysis

Methanol-fixed and RNAlater-treated data sets are combined on their mutual genes without any further data merging algorithms. DESingle (v1.6.0) was utilized to perform DE analysis between known SV cell types with default settings^87^. DESingle is specifically designed for single cell DE analysis and employs a zero-inflated negative binomial model to estimate the proportion of dropout and real zeros, allowing for a more accurate representation of differential gene expression at the single cell or single nucleus level^87^.

### Regulatory network inference

Gene regulatory network inference using single cell regulatory network inference and clustering (SCENIC) has been previously described^2,31^. It is a computational method for inferring GRN based on the expression level of transcriptional factors and their conserved motif-enriched cis-regulatory sequences. Briefly, SCENIC identifies potential gene regulatory networks by identifying regulons, defined as transcription factors and their downstream motif-enriched target genes, and by determining the activity of each of these regulons within each cell. From these analyses, a regulon activity matrix is constructed that can be utilized to cluster cells on the basis of shared regulatory networks and may identify cell types and cell states on the basis of shared activity of a regulatory subnetwork. SCENIC was implemented utilizing pySCENIC (v0.10.0). Downstream visualization and plots are created by Matplotlib (v3.2.0) and Seaborn (v0.10.0).

### Gene ontology and gene-set enrichment analysis

Gene ontology analyses and gene enrichment analyses were performed using Enrichr (https://amp.pharm.mssm.edu/Enrichr/) as previously described^2,88–91^. The combined score approach where enrichment score is calculated from the combination of the p-value computed using the Fisher exact test and the z-score was utilized. Top gene ontology (GO) terms were chosen by utilizing the combined score approach as described.

### Single-molecule fluorescent in situ hybridization (smFISH)

Fluorescent in situ hybridization was performed as previously described^2,92^ using the following RNAscope probes: *Lgr5* (Catalog No. 312171), *Epyc* (Catalog No. 572901), *Anxa1* (Catalog No. 509291), *Dpp10* (Catalog No. 553331), *Bach2* (Catalog No. 887121-C3), and *Kcnj10* (Catalog No. 458831-C3). RNAscope probes were obtained from Advanced Cell Diagnostics (Newark, CA, United States) and used with sections of cochleae from CBA/J mice at P30. Adult cochleae were dissected from the head and fixed over night at 44 °C in 4% paraformaldehyde (PFA) in 1 × PBS. Fixed adult mouse inner ears were decalcified in 150 mM EDTA for 5–7 days, transferred to 30% sucrose, and then embedded and frozen in SCEM tissue embedding medium (Section-Lab Co, Ltd., Hiroshima, Japan). Adhesive film (Section-Lab Co, Ltd., Hiroshima, Japan) was fastened to the cut surface of the sample to support the section and cut slowly with a blade to obtain thin mid-modiolar sections. The adhesive film with section attached was submerged in 100% EtOH for 60 s, then transferred to distilled water. Frozen tissues were sectioned (10 μm thickness) with a CM3050S cryostat microtome (Leica, Vienna, Austria). Sections were mounted with SCMM mounting media (Section-Lab Co, Ltd., Hiroshima, Japan) and imaged using a 1.4 N.A. objective.

## Ethical approval

All animal experiments and procedures were performed according to protocols approved by the Animal Care and Use Committee of the National Institute of Neurological Diseases and Stroke and the National Institute on Deafness and Other Communication Disorders, National Institutes of Health. All experimental protocols were approved by the Animal Care and Use Committee of the National Institute of Neurological Diseases and Stroke and the National Institute on Deafness and Other Communication Disorders, National Institutes of Health. All methods were carried out in accordance with relevant guidelines and regulations of the Animal Care and Use Committee of the National Institute of Neurological Diseases and Stroke and the National Institute on Deafness and Other Communication Disorders, National Institutes of Health.

## Supplementary information

Supplementary Information.

## Data Availability

All scripts for this study can be found in (https://github.com/Hoa-Lab/2020_Spindle-Root). All data generated in these studies have been deposited in the Gene Expression Omnibus (GEO) database (GEO Accession ID: GSE152551) and can be found on GEO [https://www.ncbi.nlm.nih.gov/geo/query/acc.cgi?acc=GSE152551]). The data has also been uploaded into the gene Expression Analysis Resource (gEAR), a website for visualization and comparative analysis of multi-omic data, with an emphasis on hearing research (https://umgear.org/p?l=58911b5d)^93^.
