# Supplementary material for: Characterization of rare spindle and root cell transcriptional profiles in the stria vascularis of the adult mouse cochlea
Source: Sci Rep. 2020 Oct 22;10:18100. doi: 10.1038/s41598-020-75238-8 (PMC7581811; doi:10.1038/s41598-020-75238-8)
Supplement: Supplementary file 1 — Supplementary Information. [file 41598_2020_75238_MOESM1_ESM.pdf]

Characterization of Rare Spindle and Root Cell Transcriptional Profiles in the Stria Vascularis of the Adult Mouse Cochlea

Shoujun Gu[1], Rafal Olszewski[1], Ian Taukulis[1], Zheng Wei[2], Daniel Martin[3], Robert J. Morell[2], and Michael Hoa[1,*]

[1] Auditory Development and Restoration Program, National Institutes on Deafness and Other Communication Disorders, National Institutes of Health, Bethesda, MD, 20892, US

[2] Computation Biology and Genomics Core, National Institutes on Deafness and Other Communication Disorders, National Institutes of Health, Bethesda, MD, 20892, US

[3] Biomedical Research Informatics Office, National Institute of Dental and Craniofacial Research, NIH, Bethesda, MD 20892, US

*corresponding michael.hoa@nih.gov

Supplementary Note

Alterations to Bioinformatic Processing Pipeline for Single-Nucleus RNA-Seq datasets.

Alterations in our bioinformatic processing pipeline from the previously published analysis [1] may have contributed to the ability to resolve rare spindle and root cells. These bioinformatic processing differences included: (1) use of Scrublet package for doublet detection and elimination, (2) use of the top 4000 highly variable genes (HVG) in this study compared to the top 2000 HVGs in the previously study likely facilitated both by the increase in number of these rare cell types captured and by the increase in number of genes detected per nuclei in the sample preservation datasets, (3) use of Leiden algorithm with resolution set in this study compared to the use of the Louvain algorithm with a resolution of 0.8, and (4) use of DEsingle [2] to perform differential expression analysis.

Cell cycle and sample dissociation effects minimally impact cluster composition in major SV cell types.

As cellular heterogeneity in gene expression can be affected by a variety of cellular processes including sample dissociation effect [3] and cell cycle status [4–6], we performed a closer examination of these factors to further establish the overall reliability of these datasets as cell type-specific transcriptome references. Our group and others have previously demonstrated that sample isolation procedures including cellular dissociation may also affect gene expression in tissue subpopulations [1,3,7]. We have previously implemented these analyses to demonstrate that single nucleus preparations demonstrate less dissociation-induced gene expression and improved cluster stability when compared to single cell preparations for RNA-Seq [1]. Cluster stability is defined as the percentage of nuclei that remain in the same cluster after dataset calibration over the total number of starting nuclei. Consistent with the previously described method, cluster stability with respect to dissociation-induced gene expression was greater than 90% for the major SV cell types (8049 of 8421 total nuclei) with venn diagrams demonstrating nuclei identified by the original clustering in purple, nuclei identified by the calibrated clustering in blue, and nuclei in the overlap of the two circles in light purple/lavender representing number of nuclei with cluster stability (Suppl. Fig. S7). SV marginal (Suppl. Fig. S7a-c) and intermediate (Suppl. Fig. S7d-f) cells demonstrated a high percentage of cluster stability. On the other hand, SV basal cells from the Ctrl dataset (Suppl. Fig. S7g) demonstrated a lower percentage of cluster stability when compared to SV basal cells in the MethFix and RNAlater datasets (Suppl. Fig. S7h and S5i, respectively).

Similar to our analysis of dissociation-induced gene expression, the effect of cell cycle-related gene expression on cluster stability was analyzed in the three datasets. Cluster stability with respect to cell cycle effect was greater than 90% for the major SV cell types (8112 of 8421 total nuclei). SV marginal (Suppl. Fig. S7j-l), intermediate (Suppl. Fig. S7m-o), and basal (Suppl. Fig. S7p-r) cells generally demonstrated high percentages of cluster stability. A similar observation was noted in the SV basal cells from the Ctrl dataset (Suppl. Fig. S7p), which demonstrated a lower percentage of

cluster stability when compared to SV basal cells from the sample preservation datasets (Suppl. Fig. S7q and S7r, respectively). Taken together, both dissociation-induced gene expression and cell cycle-related gene expression that may result from sample preparation appear to have a minimal impact on cluster composition in major SV cells in all three snRNA-Seq datasets. Thus, these snRNA-Seq datasets serve as a reliable reference for major and rare SV cell types.

Relating Transcriptional Signatures to Ultrastructural Features of Spindle Cells

Ultrastructurally, spindle cells possess numerous microvilli on their surface facing the cochlear endolymph, which increase the available surface area of these cells [8,9]. While generally both resorptive and secretory functions have been ascribed to microvilli and the SV in general, microvilli have been implicated in a variety of diverse functions including, but not limited to, regulation of substrate transport and energy metabolism, gating of ion fluxes, calcium signaling, volume regulation, mechanoreception, and light perception [10]. Luciano and colleagues have suggested that spindle cells act as a structural barrier, isolating the intrastrial compartment from the surrounding fluids in the cochlea [11]. Furthermore, Luciano and colleagues have demonstrated junctional strands between spindle cells which are populated with gap junctions which act to couple these cells to each other. Gene ontology (GO) analysis of spindle cell gene regulatory networks reveals enrichment for biological process GO terms including positive regulation of vesicle fusion (GO:0031340), regulation of calcium ion transmembrane transport (GO:1903169), regulation of calcium ion transport into cytosol (GO:0010522), regulation of cell communication by electrical coupling (GO:0010649), and regulation of potassium ion transport (GO:0043266) that connect structural features of spindle cells with their potential functions.

References

1. Korrapati, S. *et al.* Single Cell and Single Nucleus RNA-Seq Reveal Cellular Heterogeneity and Homeostatic Regulatory Networks in Adult Mouse Stria

Vascularis. *Front. Mol. Neurosci.* (2019). doi:10.3389/fnmol.2019.00316

2. Miao, Z., Deng, K., Wang, X. & Zhang, X. DEsingle for detecting three types of differential expression in single-cell RNA-seq data. *Bioinformatics* (2018). doi:10.1093/bioinformatics/bty332

3. Van Den Brink, S. C. *et al.* Single-cell sequencing reveals dissociation-induced gene expression in tissue subpopulations. *Nature Methods* (2017). doi:10.1038/nmeth.4437

4. Scialdone, A. *et al.* Computational assignment of cell-cycle stage from single-cell transcriptome data. *Methods* **85**, 54–61 (2015).

5. Nestorowa, S. *et al.* A single-cell resolution map of mouse hematopoietic stem and progenitor cell differentiation. *Blood* **128**, e20-31 (2016).

6. Tsang, J. C. H. *et al.* Single-cell transcriptomic reconstruction reveals cell cycle and multi-lineage differentiation defects in Bcl11a-deficient hematopoietic stem cells. *Genome Biol.* (2015). doi:10.1186/s13059-015-0739-5

7. Wu, H., Kirita, Y., Donnelly, E. L. & Humphreys, B. D. Advantages of Single-Nucleus over Single-Cell RNA Sequencing of Adult Kidney: Rare Cell Types and Novel Cell States Revealed in Fibrosis. *J. Am. Soc. Nephrol.* (2019). doi:10.1681/asn.2018090912

8. Anniko, M. Surface structure of stria vascularis in the Guinea pig cochlea normal morphology and atoxyl-induced pathologic changes. *Acta Otolaryngol.* **82**, 343–353 (1976).

9. Lim, D. J. & Anniko, M. Developmental morphology of the mouse inner ear: A scanning electron microscopic observation. *Acta Otolaryngol.* (1985). doi:10.3109/00016488509121766

10. Lange, K. Fundamental role of microvilli in the main functions of differentiated cells: Outline of an universal regulating and signaling system at the cell periphery. *Journal of Cellular Physiology* (2011). doi:10.1002/jcp.22302

11. Luciano, L., Reiss, G., Iurato, S. & Reale, E. The junctions of the spindle-shaped cells of the stria vascularis: A link that completes the barrier between perilymph and endolymph. *Hear. Res.* (1995). doi:10.1016/0378-5955(95)00047-8

Supplementary Figure S1

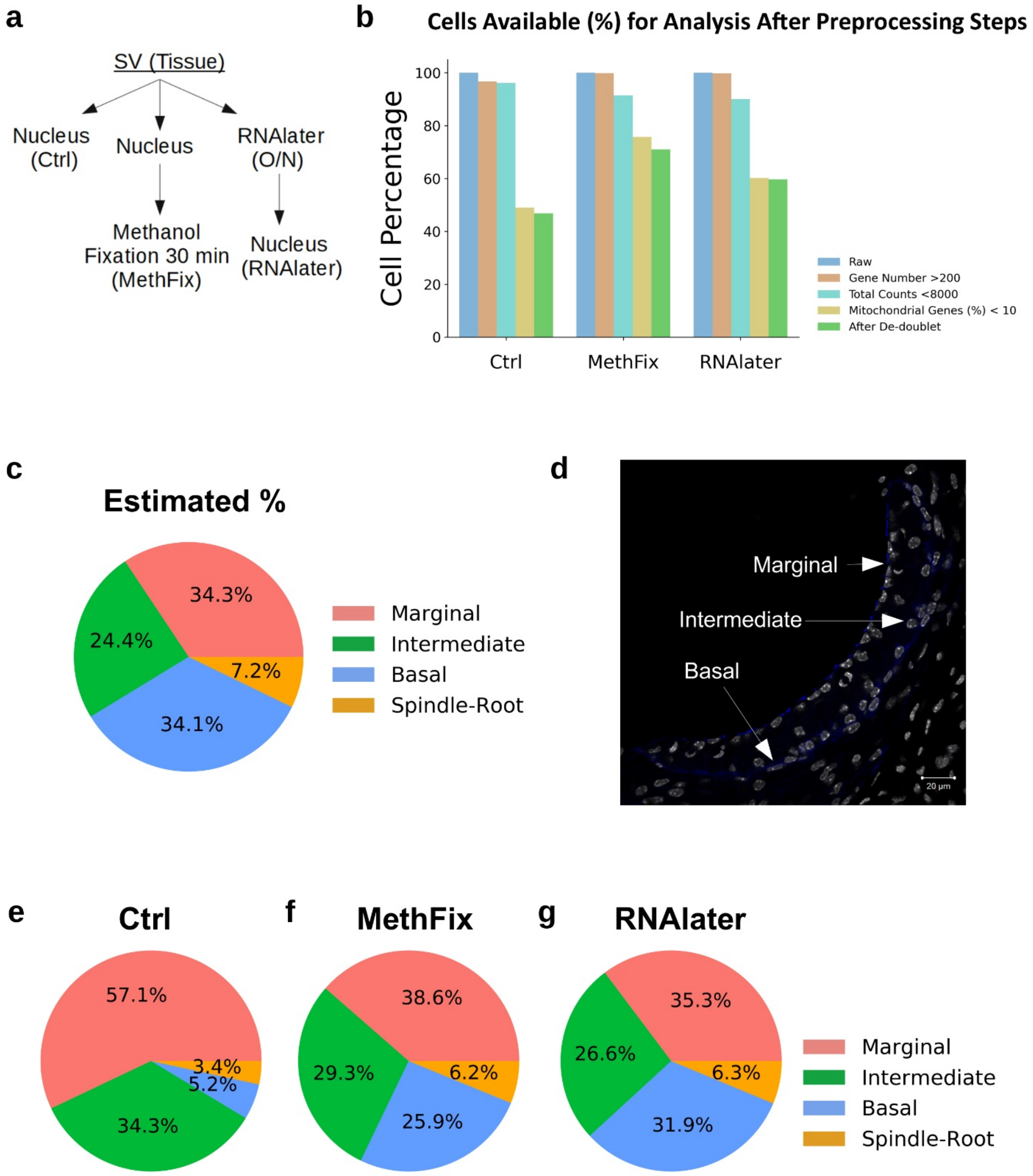

Supplementary Figure S2-1

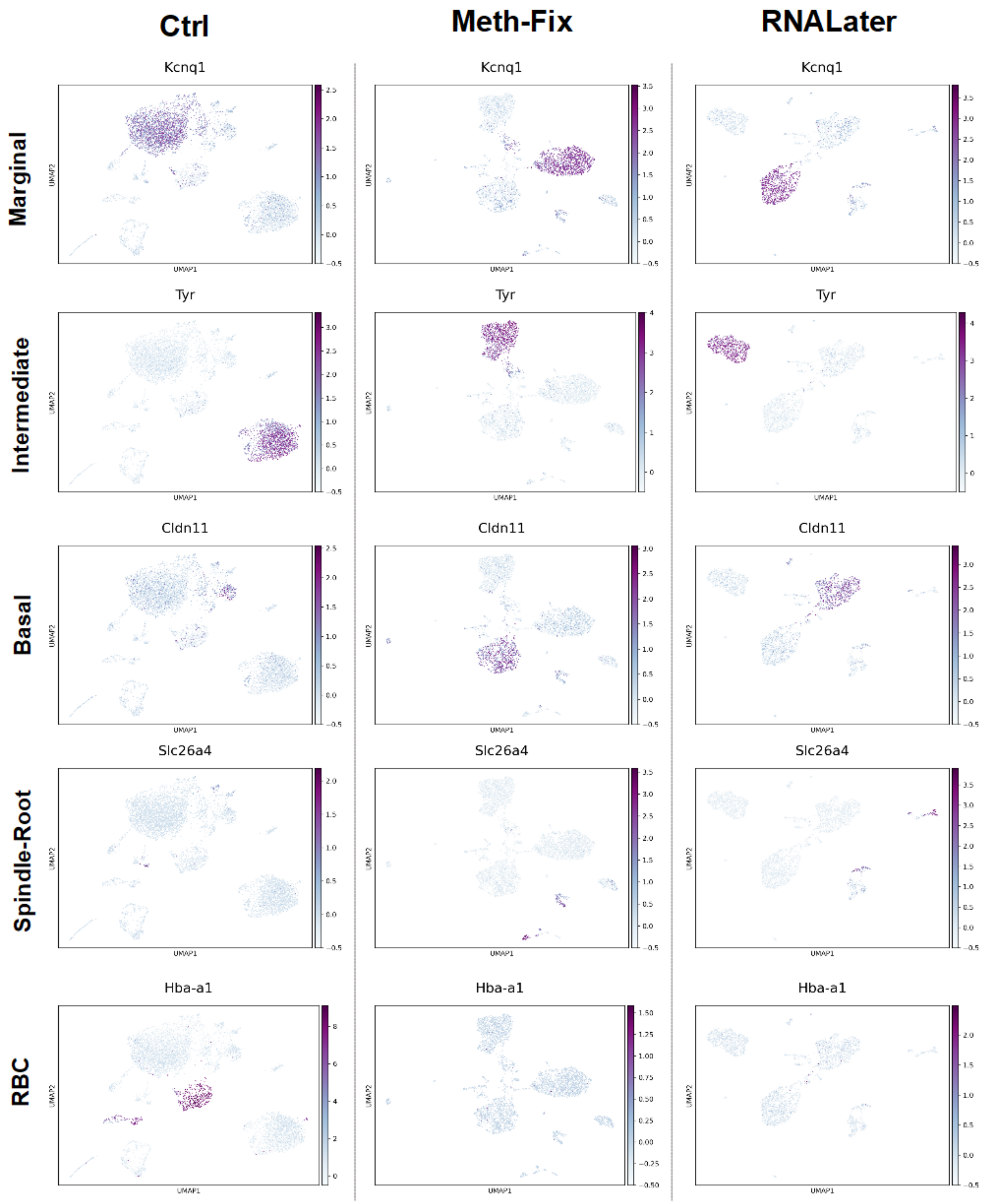

Supplementary Figure S2-2

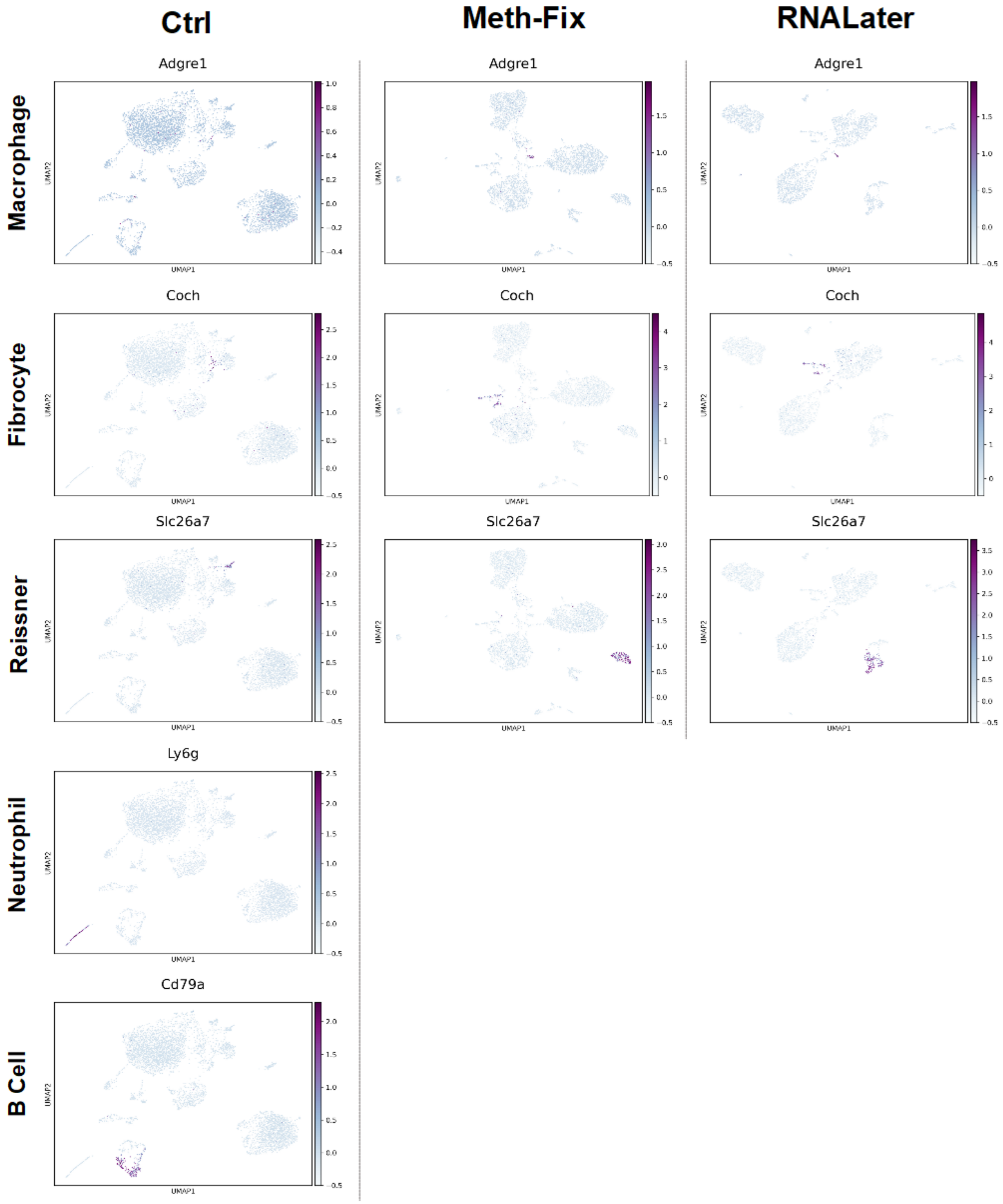

Supplementary Figure S3

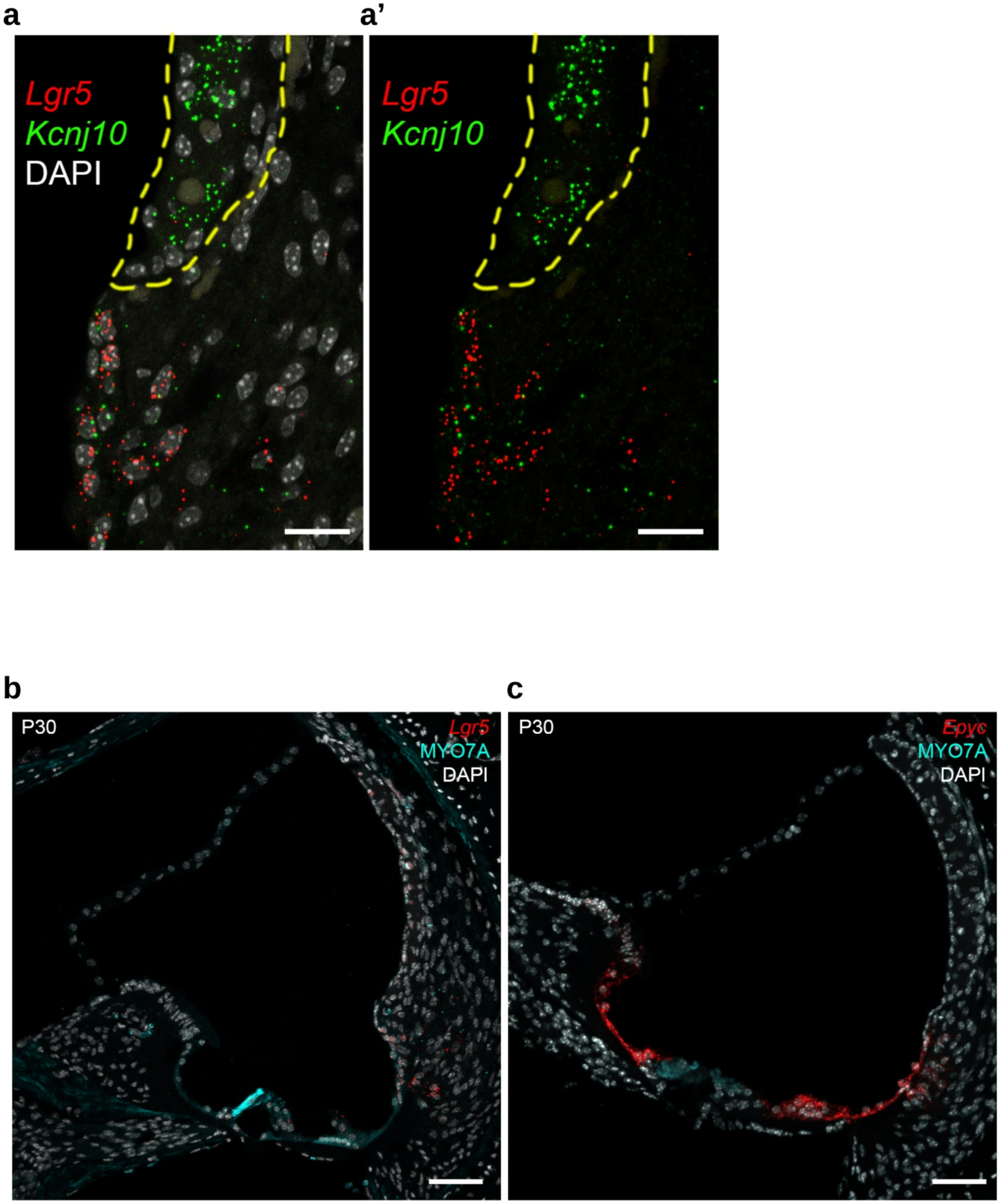

Supplementary Figure S4

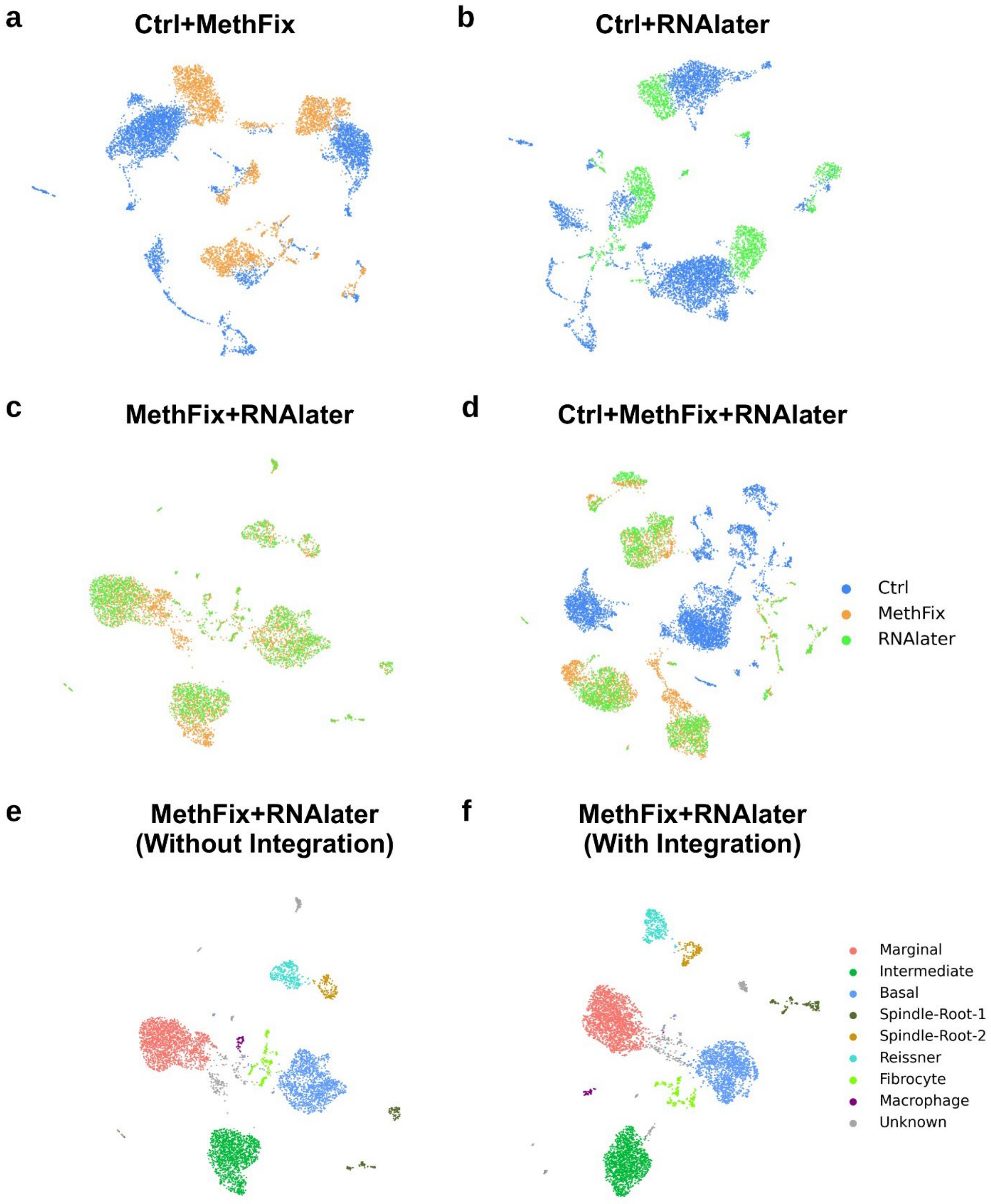

Supplementary Figure S5

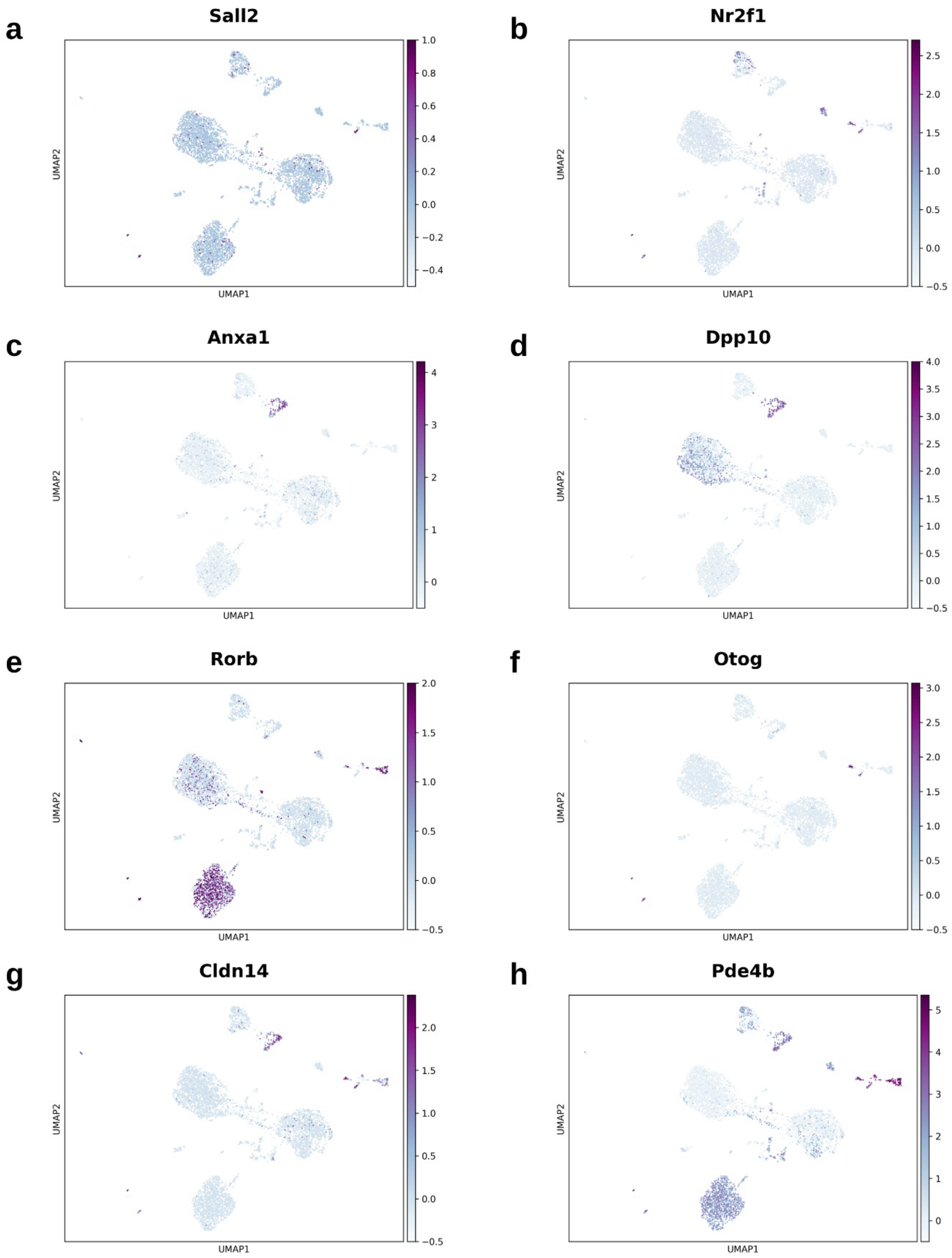

Supplementary Figure S6

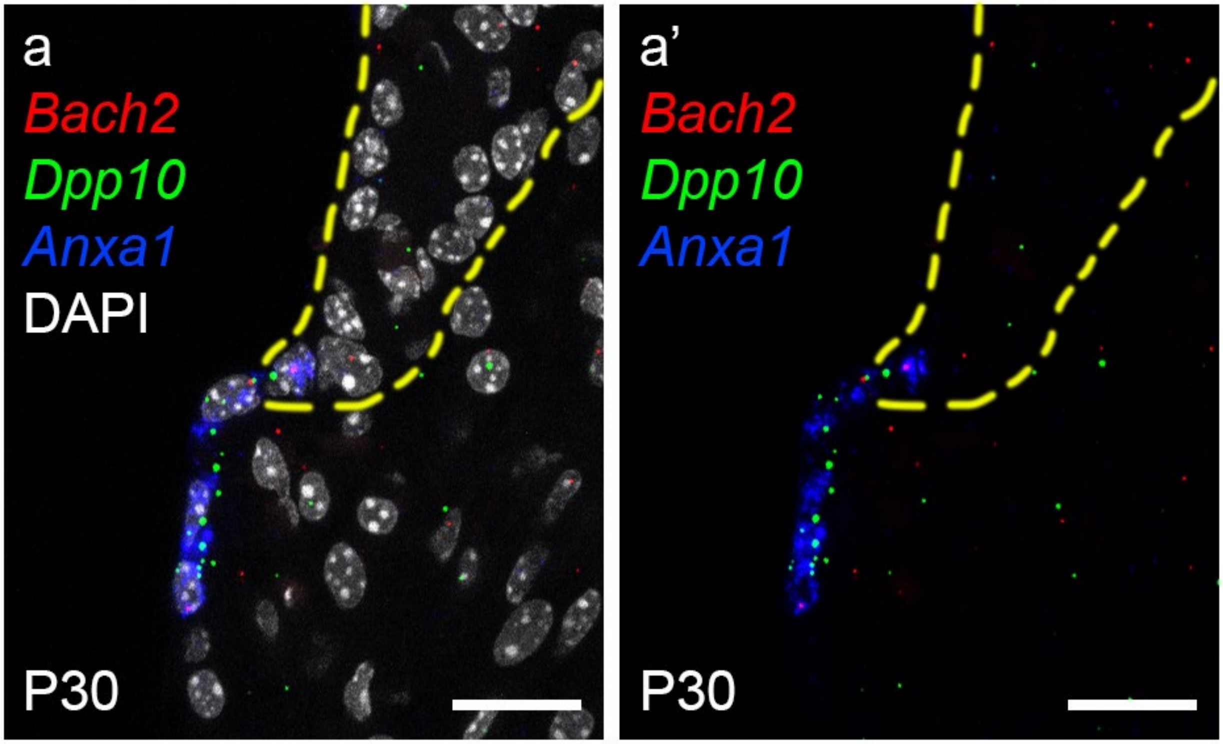

Supplementary Figure S7

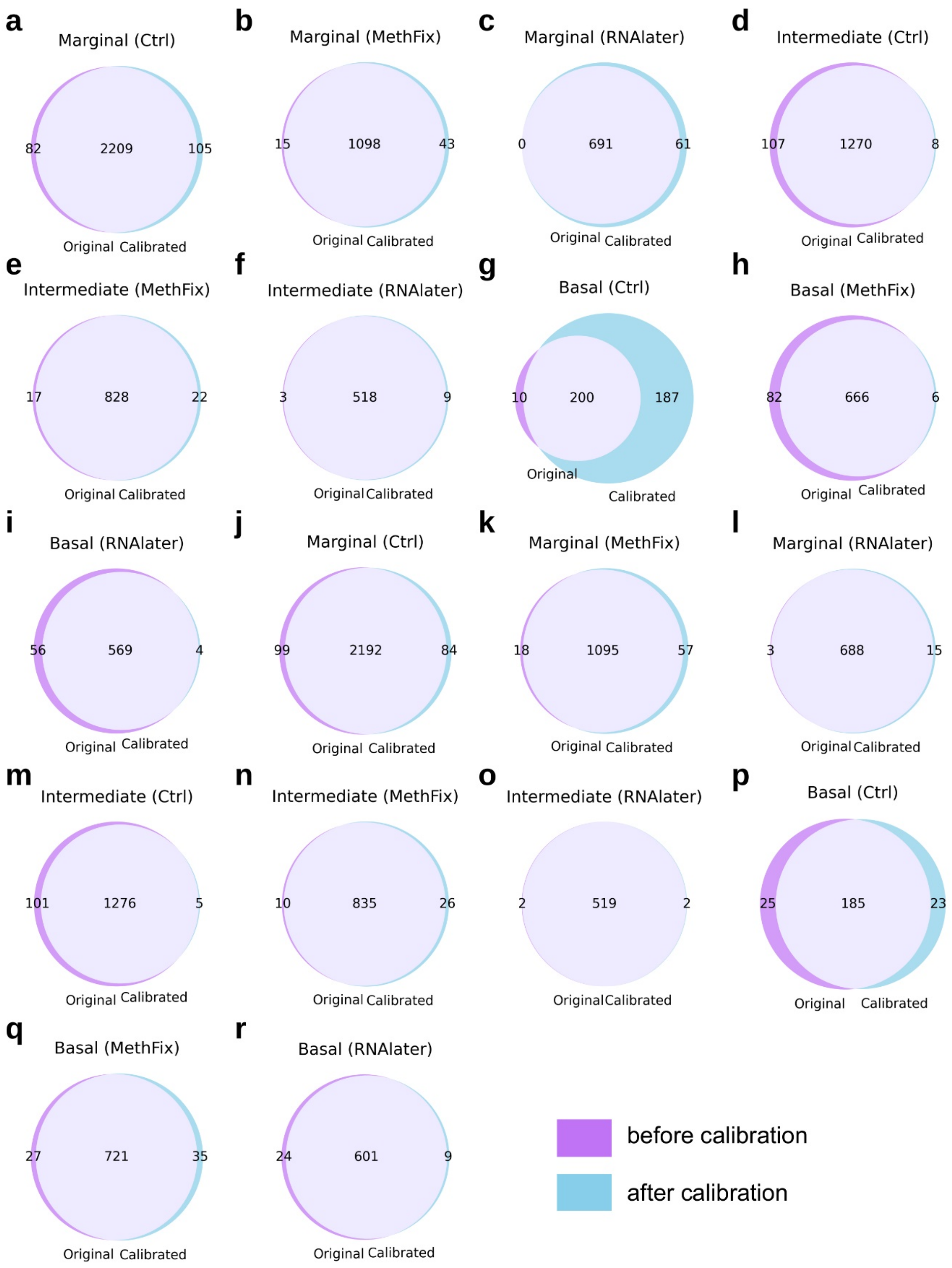

Supplementary figure legends

Supplementary Figure S1 Comparison of three datasets. (**a**) The schematic for the sample preparation steps. (**b**) Nuclei numbers after sequential data quality control steps. Based on sequential data quality control steps, 11057, 4748, and 3868 initial nuclei were downsampled to 5176, 3371, and 2310 final nuclei in the Ctrl, MethFix, and RNAlater datasets, respectively. (**c**) Estimated distributional percentage of major SV cell types from adult mice based on cell counts from midmodiolar cochlear cross-sections (N=6 adult CBA/J mice). (**d**) Representative mid-modiolar cochlear cross-section of the SV from adult mice. (**e-g**) Percentages of major SV cell types in (**e**) Ctrl, (**f**) MethFix and (**g**) RNAlater datasets.

Supplementary Figure S2-1 and S2-2 Expression of known SV cell marker genes. Normalized marker gene expression of different SV cell types in each dataset are visualized by 2D UMAP embedding on each dataset.

Supplementary Figure S3 Further validation of expression of root cell markers by smFISH. **(a-a')** Co-localization of *Lgr5* (in red) with known root cell marker, *Kcnj10* (in green), in the root cells with DAPI labeling for cell nuclei (in white) **(a)**. Yellow dotted lines indicate location of stria vascularis (SV). Note known *Kcnj10* expression in intermediate cells the stria vascularis. Same image without DAPI labeling is provided **(a')**. Scalebars are 20 μm. **(b)** In addition to expression in root cells, *Lgr5* (in red) is expressed in Deiters cells (dc) and the outer sulcus (os). MYO7A protein (in turquoise) labels hair cells. Scalebar is 50 μm. **(c)** *Epyc* expression is noted in the root cells (rc), outer sulcus (os), and greater epithelial ridge (ger). MYO7A protein (in turquoise) labels hair cells. Scalebar is 50 μm.

Supplementary Figure S4 Batch effect of three datasets. (**a**) Ctrl and MethFix datasets are combined on their mutual genes without applying any data merging algorithm. Same combinations are performed on (**b**) Ctrl and RNAlater datasets, (**c**) MethFix and RNAlater datasets and (**d**) all three datasets. (**e**) Combined MethFix and RNAlater datasets without batch correction. Clusters are annotated by SV cell type specific markers. (**f**) Combined MethFix and RNAlater datasets with batch correction by Harmony.

Supplementary Figure S5 Expression of selected regulon genes. (**a-b**) Normalized gene expression of putative *Sall2* target genes: (**a**) *Sall2* and (**b**) *Nr2f1*; (**c-d**) putative *Bach2* target genes: (**c**) *Anxa1* and (**d**) *Dpp10*; (**e-h**) putative *Rorb* target genes: (**e**) *Rorb*, (**f**) *Otog*, (**g**) *Cldn14* and (**h**) *Pde4b,* are visualized by 2D UMAP embedding on MethFix-RNAlater combined dataset.

Supplementary Figure S6 Validation of *Bach2* regulon expression. **(a)** *Bach2* (in red), *Anxa1* (in blue), and *Dpp10* (in green) RNA are expressed in spindle cells. DAPI (in white) labels cell nuclei. **(a')** Same image as (a) without DAPI labeling provided. Scalebars are 20 μm. Yellow dotted lines indicate location of the stria vascularis (SV).

Supplementary Figure S7 Minimal cell cycle and sample dissociation impact on cluster composition in sample preservation datasets. Cell cycle impact on marginal cluster composition (**a-c**), intermediate cluster composition (**d-f**), and basal cluster composition (**g-i**). Sample dissociation impact on marginal cluster composition **(j-l**), intermediate cluster composition (**m-o**), and basal cluster composition (**p-r**). Nuclei identified by the original clustering are visualized in purple. Nuclei identified by the calibrated clustering are in skyblue. Common nuclei are visualized in lavender.
